# Racism as a Determinant of Health: A Systematic Review and Meta-Analysis

**DOI:** 10.1371/journal.pone.0138511

**Published:** 2015-09-23

**Authors:** Yin Paradies, Jehonathan Ben, Nida Denson, Amanuel Elias, Naomi Priest, Alex Pieterse, Arpana Gupta, Margaret Kelaher, Gilbert Gee

**Affiliations:** 1 Alfred Deakin Institute for Citizenship and Globalization, Faculty of Arts and Education, Deakin University, Melbourne, Victoria, Australia; 2 School of Social Sciences and Psychology, University of Western Sydney, Sydney, New South Wales, Australia; 3 Australian Centre for Applied Social Research Methods, Australian National University, Canberra, Australian Capital Territory, Australia; 4 Division of Counseling Psychology, University at Albany, State University of New York, New York, New York, United States of America; 5 Oppenheimer Center for Neurobiology of Stress, David Geffen School of Medicine, University of California, Los Angeles, Los Angeles, California, United States of America; 6 Centre for Health Policy Programs and Economics, Melbourne School of Population and Global Health, University of Melbourne, Melbourne, Victoria, Australia; 7 Department of Community Health Sciences, University of California, Los Angeles, Fielding School of Public Health, Los Angeles, California, United States of America; Cardiff University, UNITED KINGDOM

## Abstract

Despite a growing body of epidemiological evidence in recent years documenting the health impacts of racism, the cumulative evidence base has yet to be synthesized in a comprehensive meta-analysis focused specifically on racism as a determinant of health. This meta-analysis reviewed the literature focusing on the relationship between reported racism and mental and physical health outcomes. Data from 293 studies reported in 333 articles published between 1983 and 2013, and conducted predominately in the U.S., were analysed using random effects models and mean weighted effect sizes. Racism was associated with poorer mental health (negative mental health: *r* = -.23, 95% CI [-.24,-.21], *k* = 227; positive mental health: *r* = -.13, 95% CI [-.16,-.10], *k* = 113), including depression, anxiety, psychological stress and various other outcomes. Racism was also associated with poorer general health (*r* = -.13 (95% CI [-.18,-.09], *k* = 30), and poorer physical health (*r* = -.09, 95% CI [-.12,-.06], *k* = 50). Moderation effects were found for some outcomes with regard to study and exposure characteristics. Effect sizes of racism on mental health were stronger in cross-sectional compared with longitudinal data and in non-representative samples compared with representative samples. Age, sex, birthplace and education level did not moderate the effects of racism on health. Ethnicity significantly moderated the effect of racism on negative mental health and physical health: the association between racism and negative mental health was significantly stronger for Asian American and Latino(a) American participants compared with African American participants, and the association between racism and physical health was significantly stronger for Latino(a) American participants compared with African American participants. Protocol PROSPERO registration number: CRD42013005464.

## Introduction

Racism can be defined as organized systems within societies that cause avoidable and unfair inequalities in power, resources, capacities and opportunities across racial or ethnic groups. Racism can manifest through beliefs, stereotypes, prejudices or discrimination. This encompasses everything from open threats and insults to phenomena deeply embedded in social systems and structures [1]. Racism can occur at multiple levels, including: internalized (the incorporation of racist attitudes, beliefs or ideologies into one’s worldview), interpersonal (interactions between individuals) and systemic (for example, the racist control of and access to labor, material and symbolic resources within a society) [1–3]. Racism persists as a cause of exclusion, conflict and disadvantage on a global scale [4], and existing data suggests racism is increasing in many national contexts (e.g., [5–9]).

Racism can impact health via several recognized pathways: (1) reduced access to employment, housing and education and/or increased exposure to risk factors (e.g., avoidable contact with police); (2) adverse cognitive/emotional processes and associated psychopathology; (3) allostatic load and concomitant patho-physiological processes; (4) diminished participation in healthy behaviors (e.g., sleep and exercise) and/or increased engagement in unhealthy behaviors (e.g., alcohol consumption) either directly as stress coping, or indirectly, via reduced self-regulation; and (5) physical injury as a result of racially-motivated violence [10–15].

The first reviews on discrimination and health were conducted in the mid 1990s and were concerned largely with conceptual and methodological advancements in studying the role of racism in health disparities in the United States [16, 17]. These reviews identified less than a dozen studies that were conducted since 1983 on racial discrimination and various health outcomes, including a range of mental and physical health outcomes. They provided early indication for the adverse impacts of racism on health and called for further research on the topic. Four additional reviews were conducted in the early 2000s that included studies from 1972 onwards, consisting of samples ranging from 13 to 53 studies [18–21]. These reviews further examined the poor mental and physical health outcomes of racial discrimination mainly in the U.S, among African-American adult populations. They found consistent evidence for associations between racism and mental health outcomes, and mixed evidence regarding associations with physical health outcomes (of which blood pressure and hypertension were the main outcomes).

Two larger systematic reviews were published in 2006 and 2009, covering a combined total of 253 empirical studies, published between 1984 and 2007 [10, 22]. International in scope, these reviews focused on racism and a plethora of health outcomes, and found the strongest and most robust associations between racism and poor mental health as well as health-related behaviors. More recently, two large-scale meta-analyses focused on the relationship between discrimination more generally, and mental health [13, 23] and physical health [13]. They examined 192 and 328 studies (with some overlap) published between 1986 and 2012, of which nearly two thirds of studies examined racial discrimination. These meta-analyses found significant negative impacts of discrimination on mental health [13, 23], and a somewhat weaker, but still significant, association with physical health [13]. Additional analyses focused specifically on racism found significant associations with mental health (operationalized as well-being), self-esteem and psychological distress, and similar results for life satisfaction, anxiety and depression (though metrics for the latter three were not published) [23]. Their results regarding the adverse mental health of racism and discrimination were confirmed also by a smaller meta-analysis and another review [24, 25].

Several reviews and meta-analyses have concentrated on specific populations, such as children and adolescents, and ethnic groups, including Asian Americans, African Americans, and Latino/a Americans, with negative mental health outcomes as the main health outcome of interest [14, 15, 26–31]. These reviews were published between 1985 and 2011 and included between 20 and 62 studies, with the exception of one review that included 121 studies [14]. They have found consistent, adverse associations with mental health, while for the few that also examined physical health findings were mixed [14, 15, 26, 27].

Several recent reviews and a meta-analysis have been conducted specifically on racism and physical health outcomes, particularly blood pressure and hypertension, and cardiovascular disease [32–35]. These works included study samples ranging from 15 to 44 studies, covering the period 1984–2013. They have found mixed, and often weak, associations between racism and hypertension and blood pressure, with the exception of ambulatory blood pressure, a potential measure of stress reactivity, which has shown consistent associations with racism [34, 35].

In summary, the reviews and meta-analyses thus far have noted that self-reported discrimination is consistently related to poor mental health, but less consistently related to poor physical health. A limitation of these prior reviews, however, is that cross-sectional studies were aggregated alongside longitudinal studies (with the exception of Schmitt et al. [23] analysis of longitudinal effects for discrimination more generally). Greater attention to longitudinal analyses is required as a way to assess causality, as well as to examine the possibility of a lag between exposure to discrimination and the development of physical health problems, which some studies have previously indicated [36–38].

### Moderators

Moderators are variables that influence the nature (i.e., direction and/or strength) of the relationship between a predictor and an outcome variable [39]. Many scholars have noted the important role that moderators may play in understanding the differential health-related outcomes among individuals experiencing racism and associated stress [11, 40, 41]. Clark et al. [40] hypothesized that moderators may first influence perceiving the environmental stimulus as a type of racism, and, second, impact processes via which racism affects the individual. More recently, Williams and Mohammed [41] developed a causal model that highlights moderators such as age, socio-economic status, racial group, gender and relational status, as influencing the racism and health relationship.

Despite this theoretical interest, the empirical data regarding the influence of moderators on relationships between racism and health outcomes is currently mixed, with previous moderation analyses of participant subgroups largely inconclusive. Some studies have found no differences in the association between discrimination and health between men and women, while others have found stronger effects on the mental health of women, while still others have found the opposite [42]. In a recent meta-analysis, gender did not significantly moderate the association between discrimination and mental health, and between discrimination and physical health [13]. Similarly, ethnicity has been a significant moderator in some studies (e.g., [43]), whereas others have found no significant differences across ethnic groups (e.g., [44]). A meta-analysis, which conducted an analysis specifically on racism and mental health, found that effects were significantly smaller for studies of anti-White discrimination compared with studies of discrimination against other ethnic groups, but found no additional differences between other ethnic groups [23]. A meta-analysis comparing the associations between discrimination and mental and physical health in Asian, Black, Hispanic, Native American, and White participants showed no significant differences based on ethnicity [13]. Similarly, little is known about age as a possible moderator of this relationship. In a meta-analysis on discrimination and mental health, age was a significant moderator in multivariate (but not univariate) analysis [23], with larger effects found for children compared to adults but no significant differences between adolescents and adults.

Characteristics of racism exposure measurement, such as the instrument name, exposure type (i.e., direct/indirect), and exposure timeframe have yet to be examined in a meta-analysis while the location where studies were conducted, publication status (i.e., published/unpublished) and year, and study sampling procedures are under-explored as moderators.

### Study rationale

This meta-analysis reviews the literature to-date focusing on the relationship between reported racism and mental and physical health outcomes. We examine key characteristics of this literature, including study methods, participant characteristics, racism exposure instruments, and health outcomes. We examine the overall magnitude of the relationship between racism and health for individual health outcomes (e.g., depression, anxiety), as well as for broader health outcome groups (e.g., negative mental health). Finally, we also examine possible study-level and participant-level moderators of the relationship between racism and health.

Despite a growing body of epidemiological evidence in recent years documenting the health impacts of racism, the cumulative evidence base has yet to be synthesized in a comprehensive meta-analysis that exclusively considers racism as a determinant of health. To our knowledge, associations between racism and some specific physical and mental health outcomes have yet to be examined in a meta-analysis focused specifically on racism (rather than on discrimination more generally) and on its associations with each of these outcomes, including general health, overweight and related conditions (e.g., obesity, diabetes), somatization, psychological stress and post-traumatic stress (PTS) and stress disorder (PTSD). Additionally, analyses focused on the association between racism and depression, anxiety and life satisfaction have been conducted [23], but the metrics of their results have yet to be published. We address these gaps in the current evidence base. Given the large number of studies examining the relationships between racism and each of these outcomes, moderation analyses per each outcome group are feasible.

Various moderators have yet to be explored in a meta-analysis of racism and health.

As well as racism resulting in ill-health, illness may also heighten the perception and reporting of racism [37]. Accordingly, it is critical to examine the longitudinal associations between racism and health, in order to better elucidate these causal pathways. A comparison of findings from longitudinal data and cross-sectional data has been conducted for general discrimination across mental health outcomes, where associations were significant for both data types. Cross-sectional data demonstrated significantly stronger effects while longitudinal effects decreased over time [23]. For the first time, this review compares these data types in the specific context of racism.

## Methods

This meta-analysis follows the reporting guidelines and criteria set in Preferred Reporting Items for Systematic Reviews (PRISMA) [45]. A protocol for this meta-analysis has been previously published [46].

### Search strategy

The literature search for this study was conducted in English, and included published and unpublished papers from the earliest time available in specified databases to the end of September 2013. Papers in languages other than English were excluded. Journal articles, theses and dissertations, books and book chapters and evaluation reports were all considered for inclusion. Other materials, including conference papers and presentations, were excluded. The majority of articles were identified through an online search. The search covered the following databases and electronic collections: Medline, PsycInfo, Sociological Abstracts, Social Work Abstracts, ERIC, CINAHL, Academic Search Premier, Web of Science and ProQuest (for dissertations/theses). For a list of search terms used, please see S1 Appendix. In addition, the authors’ personal databases were searched for additional references. We also identified 25 major literature reviews, meta-analyses and other relevant works for which reference lists were manually searched for additional articles.

### Inclusion criteria

Articles were considered for review if they consisted of empirical studies reporting quantitative data on the association between racism and a health outcome/s. The titles and abstracts of articles were first screened, and then their full texts were screened in an additional stage. We use ‘articles’ throughout this paper to refer to both published and unpublished material, while we use ‘study’ to relate to unique research; therefore one study can be reported in multiple articles, and an article may report several studies.

#### Exposure

Reported racism is the exposure examined in this study, and includes: self-reported racism experienced directly in interpersonal contact; racism directed towards a group (e.g., based on ethnicity/race/nationality) of which the person is a member; vicarious experiences of racism (e.g., witnessing racism experienced by family members or friends); proxy reports of racism (e.g., a child’s experiences of racism as reported by their parent); and internalized racism (i.e., the incorporation of racist attitudes and/or beliefs within an individual’s worldview). Exposure measures include discrimination, maltreatment, prejudice, stereotypes, aggression and related terms (see S1 Appendix), where the reason/s for these include race, skin color, ethnicity, culture, ancestry, origin, birth country, nationality, migration status, religion, language and/or accent.

More general measures of discrimination, wherein the specific effect of racism cannot be isolated, were excluded. For example, papers that used the Everyday Discrimination Scale as a measure in its original (general discrimination) format, were excluded, unless the scale was modified to explicitly specify race, skin color, ethnicity, etc. as the reason for discrimination. In cases where the majority of items assessed racism specifically and all remaining items were about discrimination broadly defined (where the reason for discrimination was not specified), the measure was included.

Exposures that focused solely on discrimination due to other reasons (e.g., gender, socio-economic status) were excluded. Several instruments combine racism and possible health outcomes in the same measure. These were excluded since this review is focused on studies in which exposure and outcome were clearly delineated as separate constructs, to allow an examination of their association without possible confounding. Exposures to race-related stress or discrimination-distress (e.g., the extent to which racial discrimination was stressful/upsetting, as measured for example by the Index of Race-Related Stress—Brief Version (IRRS-B); [47] or by the Racism Experiences Stress Scale (EXP-STR); [48]), and other exposures relating racism to health within the same instrument, or combining racism with responses to racism (e.g., how much respondents are bothered by racism) were therefore excluded. For example, versions of the Perceived Racism Scale (PRS; [49]) that included measurement of emotions, coping behaviors, and cognitive appraisals related to racist encounters, were excluded (e.g., [50]). Ecological exposure measures of racism (e.g., racial segregation), experimental exposures (e.g., videos, vignettes, tasks) (e.g., [51, 52]) and other exposures where racism was assessed by the researcher, were also excluded due to our focus on observational studies examining racism perceived by research participants.

#### Outcomes

The following health outcomes were included: (1) negative mental health (depression, anxiety, distress, psychological stress, negative affect, post-traumatic stress (PTS) and post-traumatic stress disorder (PTSD), somatization, internalizing, suicidal ideation/planning/attempts, other mental health symptoms such as paranoia and psychoticism, and general mental health); (2) positive mental health (self-esteem, life satisfaction, control and mastery, wellbeing, positive affect); (3) physical health (blood pressure and hypertension, overweight-related measures, heart conditions and illnesses, diabetes, high cholesterol, and miscellaneous/mixed measures of physical health); and (4) general health (including both physical and mental health, or unspecified as physical and/or mental health; e.g., feeling unhealthy).

Several articles used relevant exposure and outcome measures but did not examine and/or did not report their association, and were therefore excluded. Studies that measured racism as an outcome (rather than as an exposure) were also excluded.

### Screening

Approximately 20,672 articles were screened for titles and abstracts. Online database searches yielded 19,292 articles, searches of the authors’ personal databases yielded 896 articles, and another 484 articles were found in the reference lists of 25 meta-analyses and literature reviews. Search results were imported into Endnote X5 [53], duplicates deleted, and two reviewers independently screened all titles and abstracts to assess eligibility for inclusion. All authors of the study protocol [46] were involved in the screening. Articles were further considered for inclusion if their title and abstract indicated that they may contain empirical research, and that they report quantitative data on the relationship between racism and health. Articles examining discrimination more generally were retained at this stage, and their full-texts further examined in the next stage. Articles not focused primarily on the association between racism and relevant health outcomes but nonetheless reporting association/s between them were included (e.g., associations between racism and health reported as part of a large correlation matrix in a study primarily focused on other variable/s). Disagreements were resolved by consensus or by a third reviewer. After completing the first screening stage of titles and abstracts, a total of 1,110 articles were retained.

Full texts of potentially eligible articles were obtained, with each full text then independently screened by two reviewers. Disagreements were resolved by consensus or by a third reviewer. The main reasons for exclusion were the reporting of irrelevant exposure and/or irrelevant outcome measures, especially articles only reporting general experiences of discrimination. Associations with outcome groups such as health behaviors/risk behaviors, pregnancy and birth outcomes and health care utilization, were excluded as beyond the scope of this meta-analysis. Where the exact same journal article was published multiple times, only the most recent version was retained and duplicates excluded. Duplicate articles by the same authors that report identical association data were excluded. Multiple articles reporting identical association data but written by different authors were retained and their associations were averaged using Comprehensive Meta-Analysis software version 2.0 (CMA, see below) [54]. After completing the second stage of screening, 534 articles remained.

In the third and final screening stage, one reviewer examined whether articles reported appropriate and sufficient association data to allow the calculation of correlation coefficients. Decisions regarding exclusion were discussed with a second reviewer. Articles reporting only adjusted associations between racism and health were recorded but excluded from the meta-analysis. While it is appropriate to adjust for covariates in individual studies, since articles on racism and health often adjust for different sets of covariates, the effects of each covariate or sets of covariates cannot be determined. Articles adjusting for different covariates and reporting effect size data that need to be converted pose an additional limitation since such consistent conversions are not possible [13]. Given that few articles adjust for the same sets of covariates (e.g., [13]) we opted to focus solely on unadjusted effect size data.

The correlation coefficient was the most commonly reported measure of association between racism and health (particularly mental health), and was used as the measure of effect size. Beside correlations and sample sizes, standardized beta coefficients in unadjusted (univariate) regression models, which are equal to correlation coefficients, were used as correlations. All other metrics were converted to correlation coefficients. The following data formats were converted using CMA: 1) Odds Ratios (OR) and confidence intervals (CIs); 2) Means, standard deviations and sample sizes of two groups (racism and no racism); 3) Cross-tabulations (2x2) of events and non-events (racism/no racism and poor/good health); 4) Means and samples sizes for two groups, and an independent group t-value; 5) Means and samples sizes for two groups, and an independent group p-value; 6) Standardized mean differences (Cohen’s d) and sample sizes for two groups; and 7) *p*-value and sample size for correlation coefficient. In few cases (less than 2% of associations) only the sample size and *p*-level were reported, as well as whether the association was significant or not. Where the *p*-value was not significant and its exact value not reported, the correlation coefficient was conservatively recorded as zero (see [34] for a similar approach). Where the *p*-value was significant and its exact value not reported, the *p-*value was conservatively recorded just below the *p*-level (e.g., a significant *p*-value at 0.05 was recorded as *p* = 0.049999). Unstandardized regression coefficients were first converted into standardized betas and then converted into Cohen’s d using the Campbell Collaboration web-based effect size calculator [55, 56], before their conversion into correlations. Covariances were converted into correlations using corresponding standard deviations [57]. When the sample size was described as a range, the range’s minimum sample size was used.

**Fig 1:**
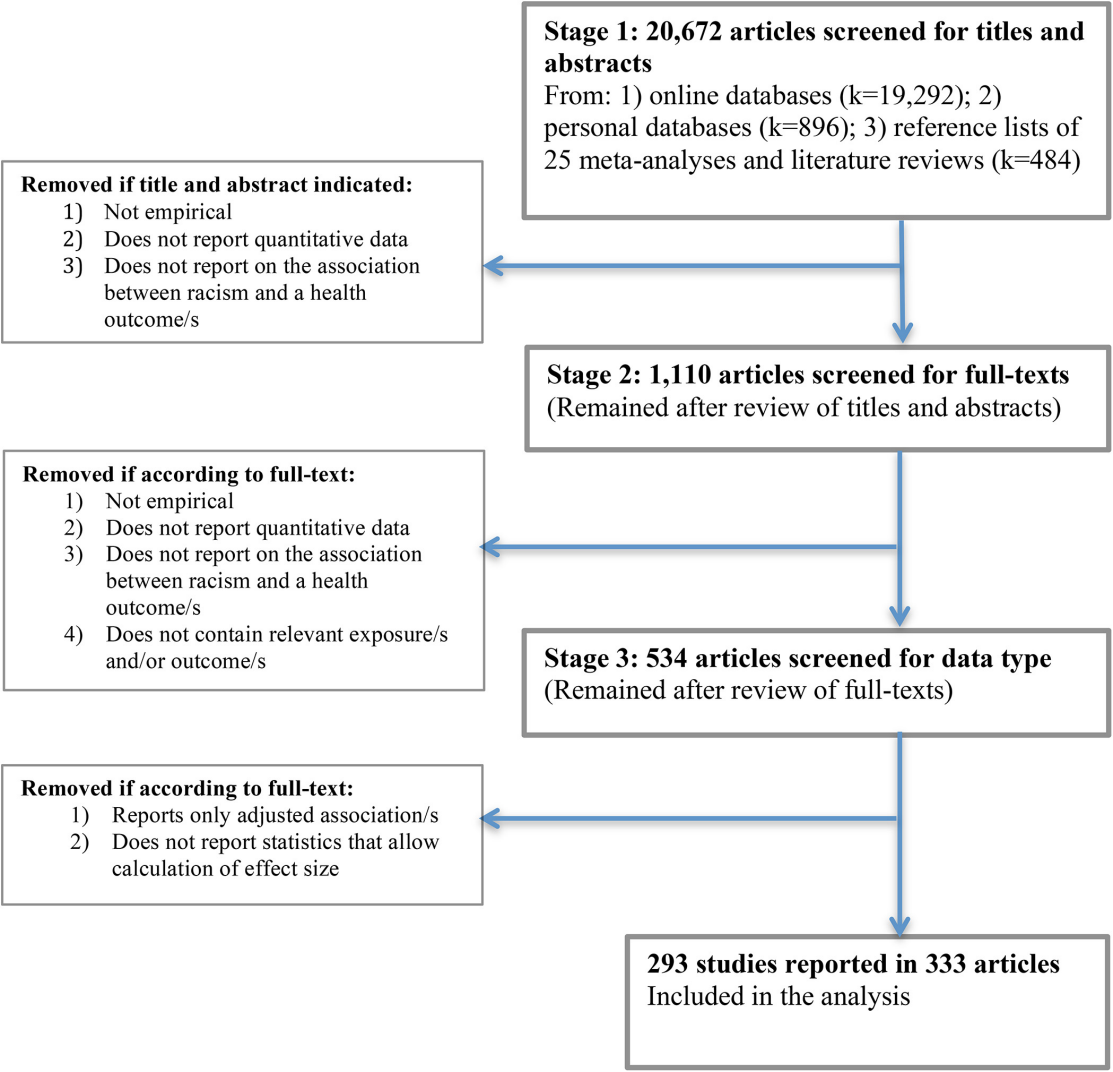
PRISMA screening process flowchart.

After exclusion of articles that did not report appropriate and sufficient association data to allow the calculation of correlation coefficients (including articles reporting adjusted effect size data only), 333 articles were left that comprised the final sample for analysis. Fig 1 summarizes the numbers of articles included at each stage of screening.

### Data extraction and coding

Articles were reviewed and data were extracted and coded by six reviewers, including two of the authors and four postgraduate students (three doctoral students and one master’s student, all with experience in research on racism and health). An Excel spreadsheet and a corresponding manual were developed for data extraction purposes (both available from the authors upon request). Reviewers extracted five types of data from each article, including data at the level of the study, participants, exposure measures, outcome measures, and effect size data. Before extraction began, reviewers read the study protocol [46], and previous meta-analyses on the topic. They were instructed on using the data extraction manual and spreadsheet, practiced extracting data from several articles, and discussed unclear issues with the first and second authors. Two reviewers each independently extracted data from a random sample of approximately 10% of articles, for which agreement was 85%. Data from all other articles were extracted by one of the six reviewers, with another reviewer double-checking coding. Disagreements were resolved by a third reviewer.

### Data integration

Using CMA, effect size data were coded so that a negative correlation indicates association between high levels of racism and low (i.e. poor) levels of health, that is, when racism increases, decreased health was coded as a negative association (or OR values lower than 1). Where OR values higher than 1 originally indicated association between high levels of racism and poorer health, and values lower than 1 originally indicated association between high levels of racism and better health, these values were reverse-coded using 1/OR.

Weighted effect sizes were calculated to account for variation in sample sizes, giving more weight to effects from larger samples. When a study comprises multiple associations between racism and health for the same participant group/s, these associations are not independent. To ensure that data are independent, each study should contribute exactly one association per analysis. A single association can be selected or calculated through averaging. We chose averaging as it allows retaining as much data as possible. We extracted all relevant associations and used a shifting unit of analysis approach (e.g., [58]) to conduct analyses both at the level of individual outcomes (e.g., for depression only), and at the level of broader outcome groups (e.g., negative mental health), in which case we averaged associations for different negative mental health outcomes such as depression, anxiety and distress. Where multiple articles reported associations from the same study or data source, these associations were averaged. There were 25 such studies, reported in 70 articles. Studies utilized in multiple papers included the following: Family and Community Health Study (FACHS; k = 11), Coronary Artery Risk Development in Young Adults Study (CARDIA; k = 4), United for Health Study (k = 4), Aboriginal Birth Cohort Study (ABC; k = 3), California Health Interview Survey (CHIS; k = 3), Diabetes Study of Northern California (DISTANCE, k = 3), International Comparative Study of Ethnocultural Youth (ICSEY; K = 3), Latino Acculturation and Health Project (k = 3), National Survey of American Life (NSAL; k = 3), Black Women's Health Study (BWHS; k = 2), Detroit Arab American Study (DAAS; k = 2), Maryland Adolescent Development in Context Study (MADICS; k = 2), National Latino and Asian American Study (NLAAS; k = 2), National Survey of Black Americans (NSBA; k = 2), Oslo Health Study (k = 2), and Sinai Improving Community Health Survey (k = 2). We also examined associations from papers by the same first author where the names of data sources were not mentioned but where the methodology and samples characteristics were identical or nearly identical, suggesting the same data may have been used in multiple papers. Nine such potential data sources, each reported in multiple papers, were identified in discussion between two reviewers. Associations were averaged for each data source using CMA.

We used the random effects model in aggregating effect sizes in all analyses. This model has been used in previous meta-analyses on the topic, as it is more appropriate than a fixed-effects model given various differences in methods, instrumentation and sample characteristics across studies (e.g., [13]) and considering that our aim is to generalize findings to the population of studies on racism and health outcomes (see also [31, 59]). Mixed effect models were used in all moderator analyses, as a more conservative approach that enables testing of differences between different moderator levels (e.g., [31]).

### Moderation analyses

Moderation analyses were conducted separately for each individual outcome, as well as for the broader outcome groups of negative mental health, positive mental health, physical health, and general health. Moderation analyses were conducted only when at least two levels of the moderator included five or more studies. This threshold was used in prior meta-analyses, and based on minimum thresholds established in the literature [60, 61]. Study was the unit of analysis utilized in moderation for the following variables: publication status (published/unpublished), sampling procedure (representative/non-representative), country (U.S./Other than U.S.), publication year (2005 or earlier/2006 or later), and data types (cross-sectional/longitudinal). Missing values were less than 5% and excluded from all analyses.

We also conducted study-level analyses for exposure measure characteristics: exposure instrument name (comparing the 8 most frequently reported instruments, e.g., Schedule of Racist Events (SRE), Experience of Discrimination (EOD)), exposure instrument type (direct/indirect exposure to racism), exposure instrument number of items (8 or less/9 or more), exposure instrument reliability coefficient (Cronbach’s alpha) value (lower than 0.8/0.8 or higher) [62], exposure timeframe (less than 3 years/3 years or more/not specified). Categorizations were based on commonly used cutoff points (see for example [32, 63]), with the aim of retaining as much data as possible given low study numbers reporting each moderator level in several analyses. Where a study reported multiple levels of the same moderator (e.g., a study reporting two exposure instruments, each using a different timeframe), such studies were excluded from the moderation analysis to avoid violating the assumption of independence.

Additional moderation analyses were conducted for participant subgroups. We included in the analysis studies entirely focused on a single participant subgroup (e.g., females only) as well as studies that reported separate, independent associations for multiple different subgroups (e.g., associations for females and males). Participant groups analyzed included: sex (male/female), age (under 18/18 or over), U.S. ethnic group (African American/Asian American/European American/Latino/a American/Native American), birth country (local-born/foreign-born), and, among student samples, current education level (primary/secondary/tertiary).

### Quality appraisal

Several moderators were also examined as indicators of study quality, namely: 1) publication status; 2) sampling procedure; 3) data type (i.e., longitudinal versus cross-sectional); 4) exposure instrument number of items; and 5) Exposure instrument reliability coefficient (Cronbach’s alpha) value. Published studies, representative sampling, longitudinal data, 9 or more items and alpha coefficient values of 0.8 or higher for exposure measures, were considered as indicators of higher study quality.

### Publication bias analyses

Three methods were used to assess publication bias among the sample of studies. First, we produced funnel plots and examined their symmetry to assess whether there was evidence of bias. Second, we used Egger’s weighted regression method [64], where the intercept’s significance was examined for statistical evidence of bias. Third, we calculated a fail-safe N to estimate the number of un-located studies with an average zero effect size required to change the results substantively [65]. The fail-safe N allows us to assess whether the effect is an artifact of bias. Where we detected publication bias, we used the trim and fill method to estimate and adjust for missing (un-reported) studies to estimate what the effect size would have been in the absence of bias [66, 67]. All tests of publication bias, as well as the trim and fill point estimates, were calculated using the CMA program.

## Results

Descriptive statistics are provided in Tables 1 and 2. The screening resulted in 333 articles that met all inclusion criteria and comprised the final sample analyzed in this study [43–44, 68–398]. The 333 articles contained unadjusted association data from 293 unique studies, after accounting for multiple articles that report the same study and for several articles (N = 5) that report two studies each. As in previous reviews and meta-analyses, this descriptive section reports on data per article rather than per study. Since multiple articles reporting the same study often examine different subsets of the data, reporting descriptive data at the level of the study was not feasible. This approach potentially double counts participants from 25 studies, each reported in multiple articles (altogether N = 70 articles), which we recognize as a potentially minor bias. Study, exposure, and outcome characteristics are presented in Table 1. All articles were published between 1983 and 2013, with their number increasing over time: 11.4% were published between 1983–2000, 21.3% between 2001–2005, 38.4% between 2006–2010, and 28.8% between 2011 and September 2013. A majority of the articles were published in academic journals (78.4%), and theses/dissertations (20.7%). Most of the research they report was conducted in the U.S. (81.4%), followed by the U.K. (2.7%), Australia (2.7%), Canada (2.1%), The Netherlands (1.8%), Finland (1.5%), Israel (1.2%), Norway (1.2%), Spain (0.9%), Portugal (0.9%), New Zealand (0.6%) and Barbados (0.6%). Research from eight other countries/regions was reported in one article each (2.4% altogether). Most articles (89.8%) reported cross-sectional data, with only 9.0% reporting longitudinal data. The rest (1.2%) were mostly quasi-experimental. Two-thirds of articles (66.7%) reported using non-representative sampling procedures, and 30.6% of articles reported using representative sampling procedures.

**Table 1:** Study, exposure, and outcome characteristics (N = 333 articles).

Variable	Groups	No. of articles reporting	% of articles reporting
**Total number of articles**		333	100.0%
**Sample size range (N participants)**	39–100	36	10.8%
	101–200	84	25.2%
	201–300	63	18.9%
	301–1,000	92	27.6%
	1,001–48,924	58	17.4%
**Year of publication**	1983–2000	38	11.4%
	2001–2005	71	21.3%
	2006–2010	128	38.4%
	2011-September 2013	96	28.8%
**Type of publication**	Academic journal	261	78.4%
	Dissertation/Thesis/Evaluation report	70	21.0%
	Book chapter	2	0.6%
**Country**	United States	271	81.4%
	United Kingdom	9	2.7%
	Australia	9	2.7%
	Canada	7	2.1%
	The Netherlands	6	1.8%
	Finland	5	1.5%
	Israel	4	1.2%
	Norway	4	1.2%
	Spain	3	0.9%
	Portugal	3	0.9%
	New Zealand	2	0.6%
	Barbados	2	0.6%
	Other (1 article per country/ region)	8	2.4%
**Sampling procedure**	Non-representative	222	66.7%
	Representative	102	30.6%
	Other/Not reported	9	2.7%
**Data type**	Cross-sectional	299	89.8%
	Longitudinal	30	9.0%
	Other	4	1.2%
**Exposure instrument name** *	Schedule of Racist Events (SRE)	34	10.2%
	Racism and Life Experience Scales (RaLES)	20	6.0%
	Experiences of Discrimination (EOD)	19	5.7%
	Perceived Racism Scale (PRS)	19	5.7%
	Everyday Discrimination Scale (EDS)**	14	4.2%
	Perceived Ethnic Discrimination Questionnaire (PEDQ)	10	3.0%
	Multidimensional Inventory of Black Identity (MIBI): public regard subscale	10	3.0%
	Nadanolitization scale	5	1.5%
**Exposure number of items** *	Single item/s	56	16.8%
	2–8 items	117	35.1%
	9 or more	162	48.6%
	Not reported	19	5.7%
**Exposure Cronbach Alpha** *	0.79 or lower	47	14.1%
	0.80 or higher	162	48.6%
	Not reported	77	23.1%
	Mixed (instruments including subscales from both levels)	10	3.0%
	Not applicable (single items)	56	16.8%
**Exposure type** *	Direct	266	79.9%
	Indirect (e.g., group, vicarious, proxy)	60	18.0%
	Internalized	10	3.0%
	Mixed (instruments including subscales from both levels)	12	3.6%
	Not Reported	4	1.2%
**Timeframe of Racism Exposure** *	Last month	12	3.6%
	Last 12 months (includes last 6)	55	16.5%
	Last >12 months and < 3 years	10	3.0%
	Last 3 years–lifetime	47	14.1%
	Not specified	210	63.1%
	Mixed (instruments including subscales from both levels)	15	4.5%
**Outcomes–negative mental health** *	Depression	124	37.2%
	Psychological stress	71	21.3%
	Distress	61	18.3%
	Anxiety	48	14.4%
	Negative affect	25	7.5%
	PTSD and PTS	16	4.8%
	Somatization	13	3.9%
	Internalizing	12	3.6%
	Suicidal ideation, planning, attempts	12	3.6%
	General mental health	12	3.6%
	Other mental health symptoms (e.g., paranoia, psychoticism)	12	3.6%
**Outcomes–positive mental health** *	Self esteem	81	24.3%
	Life satisfaction	28	8.4%
	Control/Mastery	19	5.7%
	Wellbeing	10	3.0%
	Positive affect	4	1.2%
**Outcomes–physical health** *	Blood pressure & Hypertension	24	7.2%
	Overweight (BMI, WC, WHR, overweight, obesity)	17	5.1%
	Heart conditions/illnesses	8	2.4%
	Diabetes	7	2.1%
	Cholesterol	4	1.2%
	Miscellaneous physical health	20	6.0%
**Outcomes–general health** *	General health (unspecified/ physical & mental)	30	9.0%

* Note that numbers do not add to 100% due to articles reporting multiple exposure instrument and multiple outcomes

** Includes Major Discrimination, and instruments from the Detroit Area Study.

**Table 2:** Participant characteristic (N = 333 articles).

	No. of articles reporting		% of participants
**Age**	264	Children and adolescents (under 18 only)	15.6%
		Adults (18 and older only)	84.4%
**Sex**	322	Male	40.0%
		Female	60.0%
**US Racial/ethnic groups**	271	African American	37.1%
		European American	29.6%
		Hispanic/Latin/o American	18.6%
		Asian American	9.4%
		Native American	0.7%
		Arab American	0.7%
		International students	0.4%
		Other (definitions differ across studies)	3.6%
**Birth country**	147	Locally-born	66.9%
		Foreign-born	33.1%
**Current education, student samples**	137	Primary	13.9%
		Secondary	54.7%
		Mixed primary and secondary	3.0%
		Tertiary	28.5%
**Education completed, non-student samples**	68	Less than high school/GED	18.0%
		High school	27.2%
		More than high school	54.8%

The total sample across all studies consisted of 309,687 participants (*M* = 1,057 per study, Range: 39–48,924). The sample size per article varied greatly: 10.8% of the 333 articles reported a sample of 39–100 participants, 25.2% a sample of 101–200 participants, 18.9%% a sample of 201–300 participants, 27.6% a sample of 301–1,000 participants, and 17.4% a sample of 1,001–48,924 participants.

Articles used a variety of instruments for assessing exposure to racism, with several articles using more than one exposure instrument. The most commonly used instruments of exposure to racism were the Schedule of Racist Events (SRE) (used in 10.2% of articles), Racism and Life Experience Scales (RaLES) (6.0%), Experiences of Discrimination (EOD) (5.7%), Perceived Racism Scale (PRS) (5.7%), Everyday Discrimination Scale (EDS) (4.2%), Perceived Ethnic Discrimination Questionnaire (PEDQ) (3.0%), Multidimensional Inventory of Black Identity (MIBI)–public regard subscale (3.0%), and the Nadanolitization scale (NAD) (1.5%). Most articles (79.9%) used measures of direct exposure to racism, and 18.0% of articles used indirect measures (e.g., group, vicarious, proxy-reports). Most articles used instruments that did not specify the exposure timeframe (63.1%), while a 12-month exposure timeframe was used in 16.5% of articles, and more than 3 years (including lifetime exposure) in 14.1% of articles. With regard to internal reliability of exposure instruments, almost half of the articles (48.6%) reported instruments with Cronbach’s alpha coefficients of 0.80 or higher, and 14.1% of articles reported coefficients of 0.79 or lower. Nearly half of the articles (48.6%) used instruments with 9 or more items, over a third (35.1%) used instruments with 2–8 items, and single items were reported in 16.8% of articles.

The most frequently reported mental health outcome was depression (reported in 37.2% of articles), followed by self-esteem (24.3%), psychological stress (21.3%), distress (18.3%), anxiety (14.4%). Life satisfaction was reported in 8.4% of articles, followed by negative affect (7.5%), control and/or mastery (5.7%), posttraumatic stress and posttraumatic stress disorder (4.8%), somatization (3.9%), internalizing (3.6%), suicidal ideation, planning and/or attempts (3.6%), general mental health (3.6%), wellbeing (3.0%), and positive affect (1.2%). Other mental health symptoms such as paranoia and psychoticism, were reported in 3.6% of articles. Among physical health outcomes, blood pressure and hypertension were reported in 7.2% of articles, followed by overweight-related outcomes (Body Mass Index (BMI), overweight, obesity, Waist Circumference (WC), Waist-Hip Ratio (WHR)) (5.1%), heart conditions and illnesses (2.4%), diabetes (2.1%), cholesterol (1.2%). Miscellaneous physical health outcomes were reported in 6.0% of articles. Miscellaneous physical health outcomes included: 1) measures that combine some of the physical health outcomes listed above (e.g., a measure combining blood pressure *and* diabetes); and 2) measures of other health outcomes listed below, either on their own, combined with each other, or combined with outcomes listed above. These included: angina back problems, arthritis, asthma, bodily pain, brittle bones, cancer, constipation, diarrhea, ear infection, exhaustion, fever, headache, gastrointestinal infection and disease, general/overall physical health, kidney and liver ⁄ gallbladder problems, major paralysis, muscular problems, nausea, neurological conditions, number of childhood illnesses, osteoporosis, Parkinson’s disease, physical disability, physical functioning and role-physical, physical health-related quality of life, respiratory infection, rheumatism, scabies, sickle cell disease, sickle cell trait, skin infection, sleeping problems, sore throat, stomachache, stroke, and trouble breathing. General health outcomes, either unspecified as physical or mental health, or combining physical and mental health, were reported in 9.0% of articles.

Table 2 reports the characteristics of the participants across articles. Most articles reported age, sex and, for U.S. articles, ethnic groups (Table 2). Age was reported in 264 articles, where 84.4% of participants were aged 18 years or above. Sex was reported in 322 articles, with females accounting for 60% of the total sample. Ethnic groups in the U.S. were reported in 271 articles. The largest participant subgroup was African Americans (37.1%), followed by European Americans (29.6%), Hispanic/Latino/a Americans (18.6%), and Asian Americans (9.4%). Birth country was reported in 147 articles, where 66.9% of participants were native-born, and 33.1% foreign-born. Level of education was captured in two ways. In 137 articles reporting student samples, 13.9% of participants were elementary, 54.7% were secondary, and 28.5% were tertiary school students. Additionally, in 68 articles that reported the highest level of education completed, 18.0% of participants completed less than high school/ General Education Development (GED), 27.2% completed high school, and 54.8% completed more than high school. Additional papers reported different groupings of highest levels of education that are not presented here.

### Effect sizes (*r*) by outcome groups

**Fig 2:**
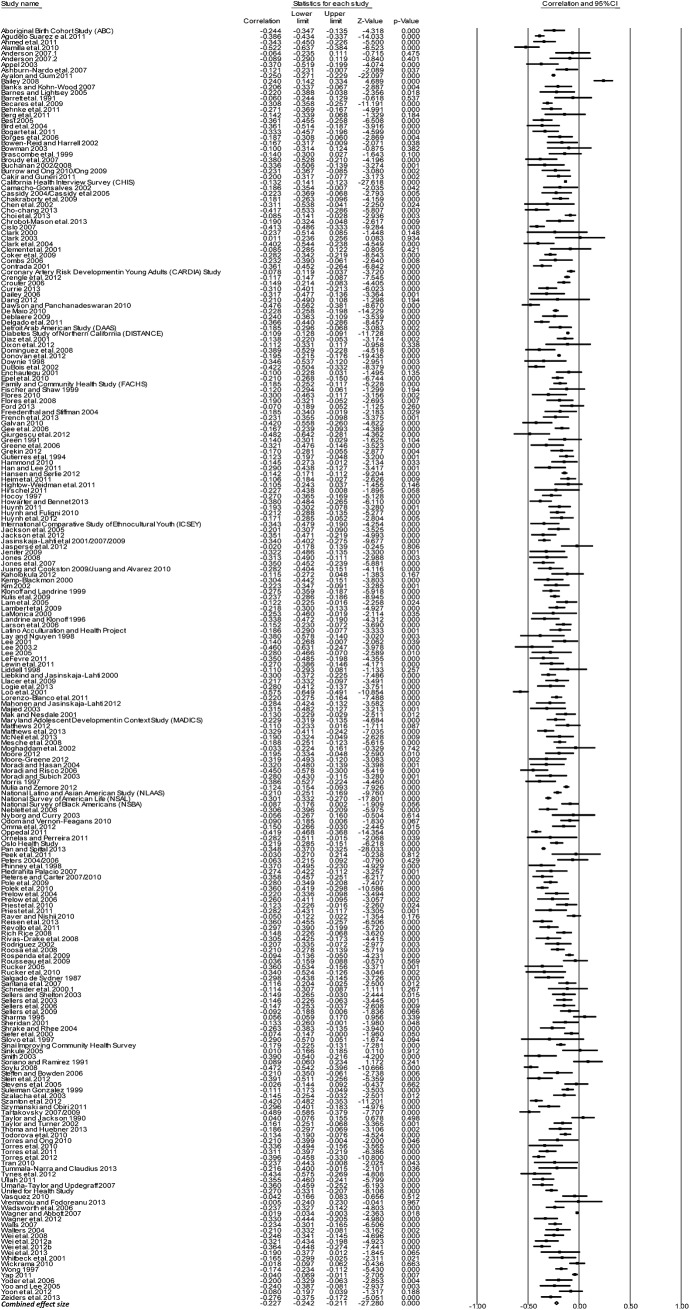
Forest plot of the effect sizes for individual studies included in the meta-analysis: Negative Mental Health (k = 227).

**Fig 3:**
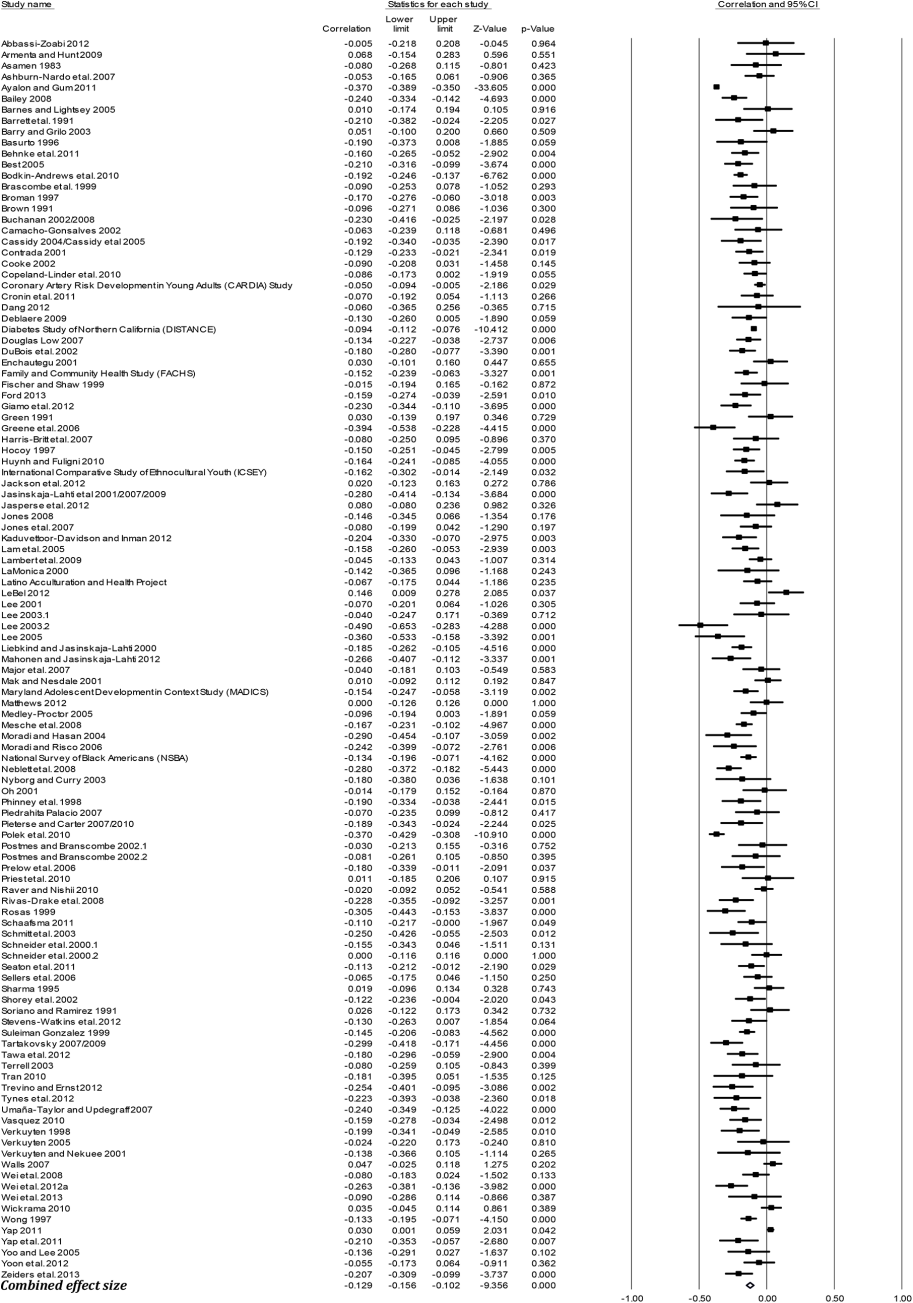
Forest plot of the effect sizes for individual studies included in the meta-analysis: Positive Mental Health (k = 113).

**Fig 4:**
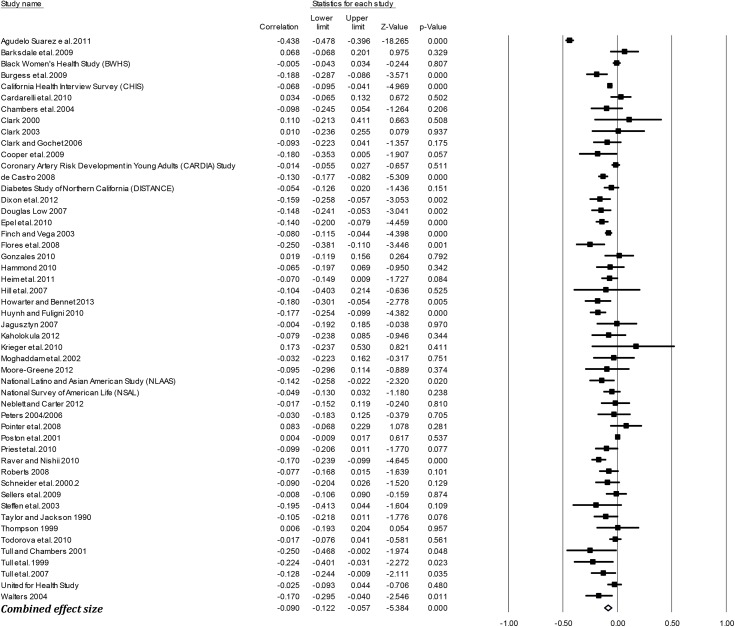
Forest plot of the effect sizes for individual studies included in the meta-analysis: Physical Health (k = 50).

**Fig 5:**
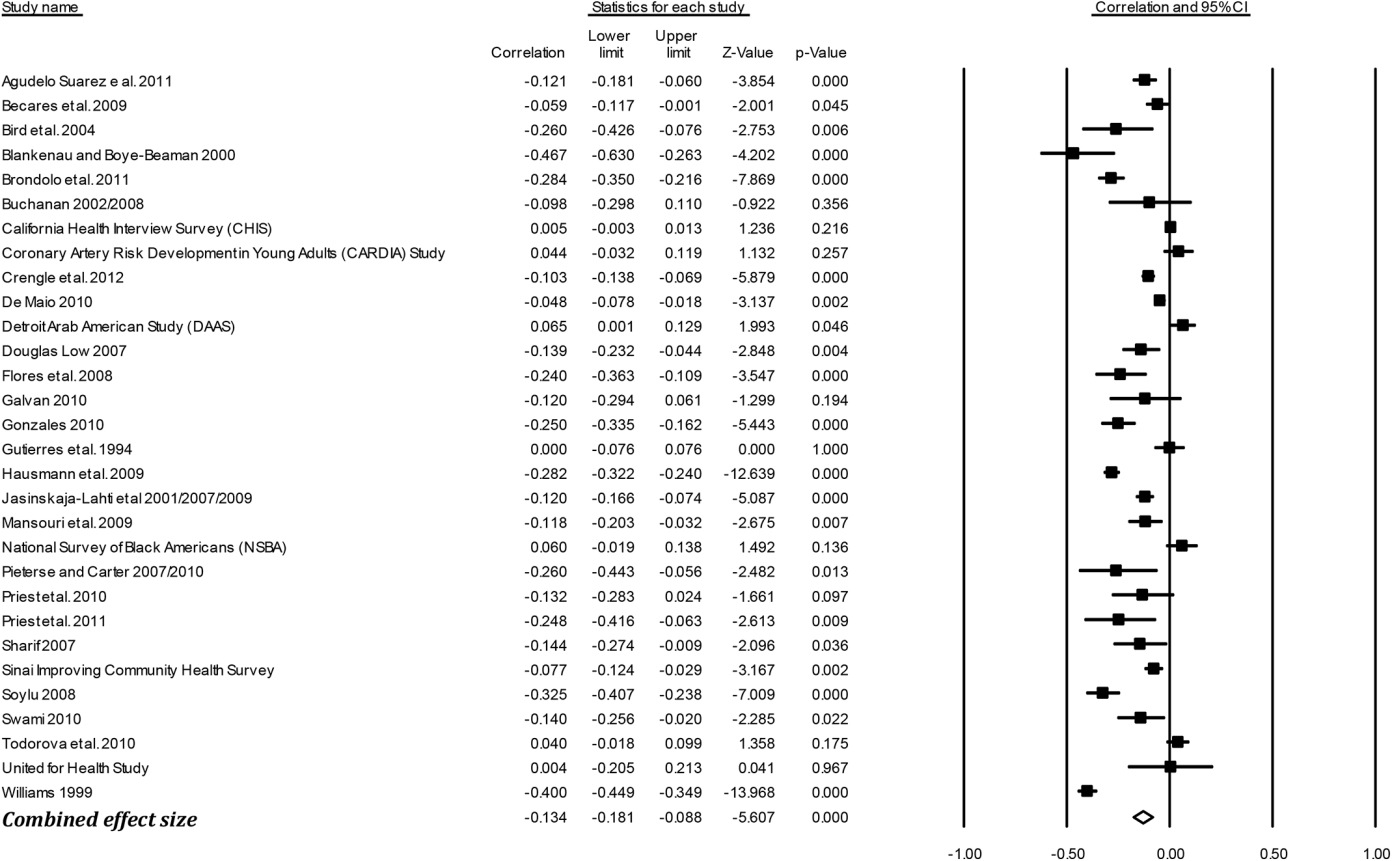
Forest plot of the effect sizes for individual studies included in the meta-analysis: General Health (k = 30).

Table 3 presents the mean weighted effect sizes for the associations between racism and negative mental health, positive mental health, physical health, and general health using a random-effects model. Study-level results and forest plots are presented for the four main outcome groups (see Figs 2–5). Of these four health outcome groups, the largest mean weighted effect size was for negative mental health (*r* = -.23, 95% CI [-.24,-.21], *k* = 227) and the smallest mean weighted effect size was for negative physical health (*r* = -.09, 95% CI [-.12,-.06], *k* = 50). The mean weighted effect size for positive mental health was *r* = -.13 (95% CI [-.16,-.10], *k* = 113), and for general health *r* = -.13 (95% CI [-.18,-.09], *k* = 30). Based on the criteria of non-overlapping Confidence Intervals (CIs), these data suggest stronger effects of racism on negative mental health compared with physical health, general health, and positive mental health.

**Table 3:** Effect sizes for associations between racism and health outcomes.

Outcome group	Outcome	r	Lower CI	Upper CI	z	p-value	k	Q-value	p-value Q
Negative mental health (NM)	DEP	-0.21	-0.23	-0.19	-18.40	<0.001	109	976.78	<0.001
	DIS	-0.22	-0.25	-0.19	-14.11	<0.001	55	447.87	<0.001
	STR	-0.27	-0.30	-0.23	-12.82	<0.001	66	891.69	<0.001
	ANX	-0.24	-0.29	-0.19	-9.50	<0.001	40	249.34	<0.001
	INT	-0.26	-0.34	-0.17	-5.65	<0.001	9	39.11	<0.001
	NA	-0.20	-0.24	-0.16	-10.00	<0.001	23	69.73	<0.001
	PTS/PTSD	-0.34	-0.40	-0.27	-8.96	<0.001	16	68.48	<0.001
	SOM	-0.23	-0.29	-0.17	-7.61	<0.001	13	40.07	<0.001
	SUI	-0.16	-0.19	-0.12	-8.57	<0.001	10	3.76	0.927
	MHS	-0.21	-0.29	-0.12	-4.72	<0.001	11	136.39	<0.001
	GMH	-0.18	-0.24	-0.12	-5.55	<0.001	12	48.84	<0.001
	Overall NM	-0.23	-0.24	-0.21	-27.28	<0.001	227	2278.70	<0.001
Positive mental health (PM)	SE	-0.12	-0.15	-0.10	-9.28	<0.001	78	284.86	<0.001
	CON	-0.11	-0.14	-0.07	-5.92	<0.001	18	40.56	0.001
	LS	-0.16	-0.22	-0.10	-5.35	<0.001	29	295.24	<0.001
	PA	0.00	-0.06	0.07	0.09	0.926	4	1.02	0.796
	WB	-0.19	-0.26	-0.12	-5.10	<0.001	10	33.89	<0.001
	Overall PM	-0.13	-0.16	-0.10	-9.36	<0.001	113	945.00	<0.001
Physical health (PH)	BP & HTN	0.00	-0.02	0.01	-0.24	0.814	24	25.78	0.312
	CHO	0.00	-0.02	0.02	-0.10	0.919	4	1.84	0.606
	DIA	-0.02	-0.09	0.04	-0.70	0.482	7	14.98	0.020
	HRT	0.00	-0.05	0.06	0.15	0.880	8	9.79	0.201
	OW	-0.08	-0.11	-0.05	-5.31	<0.001	15	22.16	0.075
	Misc	-0.13	-0.18	-0.08	-5.15	<0.001	20	251.00	<0.001
	Overall PH	-0.09	-0.12	-0.06	-5.384	<0.001	50	445.520	<0.001
General health (GH)	GH	-0.13	-0.18	-0.09	-5.61	<0.001	30	615.85	<0.001

DEP–Depression; DIS–Distress; STR–Stress; ANX–Anxiety; INT–Internalizing; NA–Negative affect; PTS/PTSD–Post-traumatic stress and post-traumatic stress disorder; SOM–Somatization; SUI–Suicidal ideation, planning, and attempts; MHS–Other mental health symptoms (e.g., paranoia, psychoticism); GMH–General mental health; Overall NM–Overall negative mental health; SE–Self-esteem; CON–Control/Mastery; LS–Life satisfaction; PA–Positive affect; WB–Wellbeing; Overall PM–Overall positive mental health; BP & HTN–Blood pressure and hypertension; CHO–cholesterol; DIA–Diabetes; HRT—Heart conditions/illnesses; OW—Overweight (BMI, WC, WHR, overweight, obesity); MISC—Miscellaneous physical health; Overall PH–Overall physical health; GH—General health (unspecified/ physical & mental)

Examination of the funnel plots showed fairly symmetric plots for all four outcome groups (available upon request from the authors). Using Rosenthal’s (1979) fail-safe *N* criterion that the value should be over five times greater than the number of studies included in the meta-analysis, the fail-safe *N* for all outcome groups were at least eight times larger than the criterion value, suggesting that the effect sizes are unlikely to be an artifact of bias. Finally, Egger’s regression intercept was statistically significant for negative mental health, physical health, and general health, suggesting potential publication bias. It was not statistically significant for positive mental health. For negative mental health, physical health, and general health, Duval and Tweedie’s trim-and-fill procedures were utilized. The number of imputed missing studies ranged from two studies (for general health) to 47 studies (for negative mental health). Imputing resulted only in minor reductions in effect sizes, which remained significant, indicating that the impact of bias is likely to be trivial. The largest adjustment was for negative mental health, adjusted from *r* = -.23 to *r* = -.18, 95% CI [-.20,-.17].

### Effect sizes (*r*) by individual outcomes

Table 3 also presents the mean weighted effect sizes for the associations between racism and individual health outcomes using a random-effects model. Due to space limitations, forest plots are not presented for individual outcomes, but are available from the authors upon request. The majority of studies examined negative mental health outcomes, with the mean weighted effect sizes ranging from *r* = -.34 for post-traumatic stress and post-traumatic stress disorders (95% CI [-.40,-.27], *k* = 16) to *r* = -.16 for suicidal ideation (95% CI [-.19,-.12], *k* = 10). All effect sizes for the negative mental health outcomes were significantly negative, indicating that racism is related to poorer mental health.

The effect sizes for positive mental health outcomes ranged from *r* = .00 (positive affect) to *r* = -.19 (wellbeing). With the exception of positive affect (*k* = 4), which did not reach significance, racism had a significant negative association with all positive mental health outcomes.

The effect sizes for physical health outcomes ranged from *r* = .00 for each of the following: blood pressure and hypertension (95% CI [-.02, .01], *k* = 24), cholesterol (95% CI [-.02, .02], *k* = 4) and heart conditions and illnesses (95% CI [-.05, .06], *k* = 8) to *r* = -.13 for miscellaneous physical health (95% CI [-.18,-.08], *k* = 20). Except for weight-related outcomes r = -.08 (95% CI [-.11,-.05], *k* = 15) and miscellaneous physical health outcomes, there was no statistically significant association between racism and physical health outcomes.

Examination of the funnel plots showed fairly symmetric distributions (available upon request from the authors) and the fail-safe *N*s were all well above the criterion values for all individual health outcomes. Of the 23 individual outcomes, Egger’s regression intercept was statistically significant for seven: depression, distress, stress, self-esteem, life satisfaction, overweight, and general health. Imputing possible missing studies for these seven outcomes resulted in either no, or very little, reduction in effect sizes, suggesting that the impact of bias is likely to be trivial. Depression (*k* = 109) had the highest number of imputed studies (i.e., 23 studies) and largest adjustment to its random-effects point estimate from *r* = -.21 to *r* = -.18, 95% CI [-.19,-.15].

### Study-level moderators

Using moderation analyses, we examined whether associations between racism and health were moderated by study-level variables. Results are shown in Table 4. Significant moderation effects were found for all moderators except the internal reliability of exposure instruments.

**Table 4:** Effect Sizes r for Moderators of Discrimination and Health Outcomes.

	DEP	DIS	STR	ANX	NA	NM	SE	LS	PM	BP	PH	GH
**Publication status**												
Published	-0.21* (84)	-0.23* (47)	-0.26* (47)	-0.24* (32)	-0.20* (17)	-0.23* (172)	-0.14* (54)	-0.16* (23)	-0.14* (80)	N/A	N/A	-0.13* (23)
Unpublished	-0.21* (23)	-0.15* (8)	-0.28* (18)	-0.25* (8)	-0.17* (5)	-0.22* (49)	-0.07* (23)	-0.18* (6)	-0.08* (31)	N/A	N/A	-0.15* (7)
Between-groups Q	0.01	1.82	0.11	0.02	0.27	0.37	7.38*	0.16	5.89*	N/A	N/A	0.11
**Publication year**												
2005 or earlier	-0.17* (29)	-0.23* (19)	-0.23* (24)	-0.16* (16)	-0.14* (6)	-0.21* (71)	-0.13* (38)	-0.13* (13)	-0.12* (49)	-0.02 (7)	-0.08* (13)	-0.20* (6)
2006 or later	-0.22* (78)	-0.21* (32)	-0.29* (41)	-0.30* (22)	-0.21* (16)	-0.23* (150)	-0.11* (38)	-0.19* (16)	-0.13* (61)	0.00 (17)	-0.09* (37)	-0.12 (24)
Between-groups Q	6.04*	0.22	2.27	7.00*	2.78	1.78	0.24	2.04	0.21	0.36	0.07	0.45
**Country**												
United States	-0.21* (94)	-0.22* (39)	-0.26* (58)	-0.23* (31)	-0.17* (17)	-0.23* (184)	-0.11* (64)	-0.17* (22)	-0.12* (94)	N/A	-0.07* (42)	-0.15* (21)
Other than United States	-0.19* (15)	-0.21* (16)	-0.29* (8)	-0.28* (9)	-0.26* (6)	-0.22* (43)	-0.18* (13)	-0.16* (7)	-0.17* (19)	N/A	-0.16* (8)	-0.10* (9)
Between-groups Q	0.73	0.04	0.13	0.73	4.33*	0.03	5.02*	0.01	2.43	N/A	1.49	1.54
**Longitudinal vs Cross-sectional**												
Cross-sectional data	-0.21* (92)	N/A	N/A	N/A	N/A	-0.22* (197)	-0.13* (66)	N/A	-0.13* (99)	N/A	N/A	N/A
Longitudinal data	-0.16* (11)	N/A	N/A	N/A	N/A	-0.16* (14)	-0.06 (9)	N/A	-0.07* (11)	N/A	N/A	N/A
Between-groups Q	2.37	N/A	N/A	N/A	N/A	5.58*	2.63	N/A	3.00	N/A	N/A	N/A
**Sampling procedure**												
Representative	-0.18* (27)	-0.20* (15)	-0.21* (19)	-0.14* (7)	-0.19* (7)	-0.20* (59)	-0.11* (11)	N/A	-0.12* (20)	-0.04 (5)	-0.10* (15)	-0.10* (12)
Non-representative	-0.23* (75)	-0.23* (38)	-0.29* (46)	-0.27* (31)	-0.21* (16)	-0.24* (158)	-0.12* (62)	N/A	-0.13* (88)	0.00 (18)	-0.08* (33)	-0.18* (17)
Between-groups Q	3.18	0.89	3.98*	4.34*	0.21	5.13*	0.09	N/A	0.04	1.36	0.21	3.35
**Exposure: type**												
Direct interpersonal	-0.23* (78)	N/A	-0.30* (43)	-0.27* (26)	N/A	-0.24* (167)	-0.14* (54)	-0.15* (21)	-0.13* (77)	N/A	-0.09* (35)	N/A
Group or vicarious	-0.17* (17)	N/A	-0.22* (14)	-0.17* (9)	N/A	-0.19* (31)	-0.07* (8)	-0.22* (5)	-0.12* (16)	N/A	-0.08* (6)	N/A
Between-groups Q	4.68*	N/A	3.2	2.03	N/A	6.58*	3.38	0.88	0.09	N/A	1.26	N/A
**Exposure: timeframe**												
3 years or less	-0.20* (25)	-0.21* (15)	-0.30* (13)	-0.21* (7)	N/A	-0.23* (45)	-0.10* (12)	-0.09* (8)	-0.10* (21)	N/A	-0.11* (10)	-0.19* (7)
More than 3 years	-0.21* (8)	-0.24* (5)	-0.33* (6)	-0.34* (9)	N/A	-0.25* (25)	N/A	N/A	-0.11* (7)	0.00 (8)	-0.08 (14)	-0.08 (5)
Not specified	-0.22* (64)	-0.23* (25)	-0.25* (41)	-0.21* (19)	N/A	-0.23* (130)	-0.13* (61)	-0.20* (16)	-0.14* (76)	-0.02 (11)	-0.09* (21)	-0.17* (13)
Between-groups Q	0.69	0.29	2.17	5.47	N/A	0.33	0.58	4.07*	1.67	1.09	1.17	2.42
**Exposure: number of items**												
8 or less	-0.21* (53)	-0.18* (25)	-0.23* (24)	-0.26* (14)	-0.18* (15)	-0.21* (105)	-0.12* (44)	-0.15* (19)	-0.13* (62)	0.00 (9)	-0.10* (20)	-0.13* (20)
9 or more	-0.22* (42)	-0.25* (23)	-0.29* (31)	-0.23* (19)	-0.24* (7)	-0.24* (91)	-0.12* (29)	-0.19* (10)	-0.13* (43)	-0.02 (10)	-0.09* (24)	-0.17* (8)
Between-groups Q	0.34	3.95*	1.67	0.35	1.45	3.38	0.04	0.73	0.05	1.01	0.10	0.66
**Exposure: Cronbach alpha**												
Lower than 0.8	-0.28* (8)	N/A	N/A	-0.26* (5)	N/A	-0.26* (22)	-0.13* (13)	N/A	-0.15* (13)	N/A	N/A	N/A
0.8 or higher	-0.23* (55)	N/A	N/A	-0.25* (20)	N/A	-0.24* (107)	-0.16* (42)	N/A	-0.14* (63)	N/A	N/A	N/A
Between-groups Q	0.82	N/A	N/A	0.03	N/A	0.23	1.10	N/A	0.05	N/A	N/A	N/A

DEP–Depression; DIS–Distress; STR–Stress; ANX–Anxiety; INT–Internalizing; NM–Overall negative mental health; SE–Self-esteem; LS–Life satisfaction; PM–Overall positive mental health; BP & HTN–Blood pressure and hypertension; PH–Overall physical health; GH—General health (unspecified / physical & mental health combined). Numbers outside parentheses are effect sizes *r*. Numbers inside parentheses are studies’ numbers *k*. N/A–Not available due to insufficient number of studies (k<5) in at least one level of the moderator.

* p < 0.

Publication status: publication status significantly moderated the association between racism and self-esteem (*Q*(1) = 7.38, *p =* .007), and between racism and positive mental health more broadly (*Q*(1) = 5.89, *p =* .015). Published studies had larger effect sizes as compared to unpublished studies.

Publication year: publication year was a significant moderator for the association between racism and depression (*Q*(1) = 6.04, *p =* .014), and between racism and anxiety (*Q*(1) = 7.00, *p =* .008). Studies published from 2006 onwards had stronger effect sizes compared to studies published before 2006.

Country: the country where the study was conducted significantly moderated the association between racism and negative affect (*Q*(1) = 4.33, *p =* .037), and between racism and self-esteem (*Q*(1) = 5.02, *p =* .025). Specifically, the effect sizes for studies conducted outside the U.S. were larger than effect sizes for studies conducted in the U.S.

Sampling: the sampling design was a significant moderator of the association between racism and the following three outcomes: stress (*Q*(1) = 3.98, *p =* .046), anxiety (*Q*(1) = 4.34, *p =* .037), and negative mental health more broadly (*Q*(1) = 5.13, *p =* .023). The effect sizes for studies using non-representative sampling were larger than the effect sizes for studies using representative sampling.

Longitudinal versus cross-sectional data: there were sufficient numbers of studies reporting longitudinal data to allow the comparison of effect sizes from cross-sectional data versus longitudinal data for two outcome groups, positive mental health and negative mental health as well as two individual health outcomes, self-esteem and depression. Type of data collected (i.e., longitudinal versus cross-sectional data) significantly moderated the association between racism and negative mental health (*Q*(1) = 5.58, *p =* .018), but did not moderate the associations for self-esteem (*Q*(1) = 2.63, *p =* .105), positive mental health (*Q*(1) = 3.00, *p =* .083), and depression (*Q*(1) = 2.37, *p =* .124). There was insufficient data to examine data type features for physical health and other outcomes.

For negative mental health, a moderation analysis of cross-sectional and longitudinal studies (regardless of time between exposure and outcome), showed that the effect size for cross-sectional data (*r* = -.22, *z* = -25.55, *p* < .001, *k =* 197) was larger than the effect size for longitudinal data (*r* = -.16, *z* = 5.84, *p* < .001, *k =* 14, *Q*(1) = 5.58, *p =* .018). Sufficient longitudinal data were reported in studies on racism and negative mental health to allow separate analyses for short-term longitudinal data (up to 1 year between exposure and outcome) and long-term longitudinal data (more than 1 year between exposure and outcome) (not reported in the table). This moderator was significant once again (*Q*(2) = 13.08, *p =* .001). The effect size for cross-sectional data (*r* = -.22, *z* = -25.55, *p* < .001, *k =* 197) was similar to the effect for short-term longitudinal data (*r* = -.21, *z* = -4.38, *p* = < .001, *k =* 5, Q(1) = 0.049, p = .826), and significantly larger than the effect for long-term longitudinal data (*r* = -.11, *z* = -3.79, *p* < .001, *k =* 7, (Q(1) = 13.082, p <. 001), which was significant nonetheless. For the longitudinal studies reporting negative mental health outcomes, we also tested the months between exposure and outcome as a potential moderator. The number of months between exposure and outcome was not a significant predictor of effect sizes (*B* = .0057, *z* = 1.57, *p* = .115, *k* = 12). Two studies reported both short-term and long-term longitudinal effects and were excluded from this supplementary analysis.

Exposure instrument type: exposure instrument type was a significant moderator for the association between racism and depression (*Q*(1) = 4.68, *p =* .031), and between racism and negative mental health more broadly (*Q*(1) = 6.58, *p =* .010). Studies using exposure instruments that measure direct exposure to racism had larger effect sizes as compared to studies using exposure instruments that measure indirect (i.e., group or vicarious) exposure to racism.

Exposure instrument timeframe: the exposure instrument timeframe significantly moderated the association between racism and life satisfaction (*Q*(1) = 4.07, *p =* .044). Studies that ask about exposure to racism in the last 3 years or less had a smaller effect size as compared to studies using exposure instruments with unspecified timeframes.

Number of exposure instrument items: the number of items in the exposure instruments significantly moderated the association between racism and distress (*Q*(1) = 3.95, *p =* .047). The effect size for studies using exposure instruments with 9 items or more was larger than the effect size for studies using exposure instruments with 8 items or less.

Internal reliability of exposure instruments: only five outcome groups (depression, anxiety, self-esteem, negative mental health and positive mental health) had sufficient studies to allow moderation analysis using internal reliability of the exposure instrument as a moderator. In these analyses, exposure instrument reliability did not significantly moderate the associations between racism and any of the five health outcomes (*Q*(1) = 0.82, *p =* .364; *Q*(1) = 0.03, *p =* .854; *Q*(1) = 1.10, *p =* .295; *Q*(1) = 0.23, *p =* .628; *Q*(1) = 0.05, *p =* .827, respectively).

Exposure instrument name (not shown in table): additional moderation analyses examining exposure instrument utilized as a possible moderator were run for depression, negative mental health, positive mental health and physical health (with insufficient studies for all other outcomes). For depression and positive mental health, exposure instrument name was not significant as a moderator (*Q*(1) = 1.01, *p =* .605; *Q*(1) = 0.21, *p =* .650, respectively). For negative mental health, the effects for PRS (*r* = -.32, *z* = -10.39, *p* < .001, *k =* 14), and PEDQ (*r* = -.33, *z* = -8.84, *p* < .001, *k =* 9) were significantly larger than the effect for RaLES (*r* = -.20, *z* = -4.18, *p* < .001, *k =* 7) (*Q*(1) = 4.44, *p =* .035; *Q*(1) = 4.40, *p =* .036, respectively). For physical health, the effects for NAD (*r* = -.11, *z* = -3.42, *p* = .001, *k =* 5), EDS (*r* = -.07, *z* = -2.61, *p* = .009, *k =* 6) and EOD (*r* = -.09, *z* = -2.17, *p* = .030, *k =* 6) were significantly larger than the effect for PRS (*r* = .00, *z* = 0.62, *p* = .534, *k =* 6) (*Q*(1) = 12.05, *p =* .001; *Q*(1) = 7.19, *p =* .007; *Q*(1) = 5.02, *p =* .025, respectively).

### Participant-level moderators

We conducted moderation analyses for participant subgroups, examining age, sex, ethnicity (for studies conducted in the U.S.), level of education and birthplace as potential moderators of the association between racism and health outcomes. Age (≥18 years vs. < 18 years), sex (male vs. female), current education level (primary vs. secondary vs. tertiary) and birthplace (local vs. foreign born) did not significantly moderate the association between racism and any of the health outcomes examined, where at least two levels of the moderator consisted of five or more studies. However, the associations between racism and depression, negative mental health, and physical health were significantly different across U.S. ethnic groups.

For depression (*Q*(2) = 6.29, *p* = .043), the associations for Asian Americans produced the largest effect size (*r* = -.28, *z* = -6.21, *p* < .001, *k =* 11), followed by associations for Latino/a Americans (*r* = -.24, *z* = -12.49, *p* < .001, *k =* 29). While these two groups did not have significantly different effect sizes from each other, they both had significantly larger effects when compared with African Americans (*r* = -.18, *z* = -8.06, *p* < .001, *k =* 38). Effect sizes for the other ethnic groups (European Americans, Native Americans) were few in number and were therefore not included in the analysis.

For negative mental health, which consists of depression as well as other mental health outcomes, the overall moderation analysis including 5 ethnic groups was not significant (*Q*(4) = 7.64, *p* = .106). However, ethnicity was significant as a moderator in pairwise analyses comparing effects for African Americans with effects for Latino/a Americans and for Asian Americans. Accordingly, the associations for Asian Americans produced the largest effect size (*r* = -.28, *z* = -8.30, *p* < .001, *k =* 20), followed by Latino/a Americans (*r* = -.25, *z* = -14.32, *p* < .001, *k =* 49). While these two groups did not have significantly different effect sizes from each other, both had significantly larger effect sizes when compared with African Americans (*r* = -.20, *z* = -10.79, *p* < .001, *k =* 68). The effect sizes for European Americans (*r* = -.21, *z* = -5.28, *p* < .001, *k =* 6), and for Native Americans (*r* = -.21, *z* = -8.25, *p* < .001, *k =* 6), were not significantly different from the effect sizes for the 3 other ethnic groups.

For physical health, African Americans and Latino/a Americans were the only groups for which sufficient numbers of associations were reported to allow moderation analysis. Ethnicity significantly moderated the association between racism and physical health (*Q*(1) = 5.22, *p* = .022). Specifically, the effect size for Latino/a Americans (*r* = -.12, *z* = -3.26, *p* = .001, *k =* 5) was significantly larger than the effect size for African Americans (*r* = -.03, *z* = -2.33, *p* = .020, *k =* 24).

## Discussion

This meta-analysis is the first to focus specifically on racism and health across a range of populations, national contexts and health outcomes. Using a comprehensive and rigorous search protocol, a total of 293 studies reported in 333 articles were located. Consistent with previous systematic reviews, trends over time indicate increasing output of published articles focusing on racism and (particularly adult) health, while a relative majority of research is still being conducted in the U.S. among African Americans [10, 14]. However, this meta-analysis indicates an increasing trend for articles to include European Americans (often for comparative purposes) with a growing focus on Latina/o and Asian Americans as well. The majority of participants were adults, and only about 16% of participants were younger than 18 years old. Most participants were female, and over a third of articles focused on student samples (predominately from secondary and tertiary, rather than elementary, education levels). This research demonstrates a primary focus on locally-born populations but with a third of articles also including migrant populations (see also [14]). Comparative studies on the impact of racism over time on both migrants and native-born populations of similar ethnic/racial backgrounds are currently lacking and should be examined in future research.

This meta-analysis indicates that racism is significantly related to poorer health, with the relationship being stronger for poor mental health and weaker for poor physical health. After adjusting for publication bias, the correlation with poor mental health remained twice as large as the correlation for poor physical health, with results for general health (unspecified as mental or physical health/mental and physical health combined) falling in-between. This contrasts with the findings reported by Pascoe and Richman [13], where the association between perceived discrimination and physical health compared to mental health did not differ significantly. One possible reason for the discrepancy is that Pascoe and Richman [13] examined multiple forms of perceived discrimination, whereas the present study focused on discrimination based explicitly on race and related classifiers like ethnicity and nationality.

A more detailed examination of health outcomes indicates a two-fold range in the strength of association between racism and poor mental health (from r = -0.16 for suicidal ideation, planning, and attempts and r = -0.34 for post-traumatic stress and post-traumatic stress disorder). After adjustment for publication bias, depression (the most commonly reported outcome) had the same magnitude of association as the broader category of negative mental health. With regard to physical health, only overweight-related outcomes and a range of miscellaneous physical health outcomes were significantly associated with racism, as found in recent longitudinal studies [399, 400]. A recent review [401] found only some significant associations between racism and obesity. Our different finding may be due to the more comprehensive nature of our examination of overweight-related outcomes (including BMI, WC, WHR, overweight and obesity), the inclusion of additional studies (some of which were conducted more recently), and the use of different designs (a meta-analysis versus a literature review).

Small sample sizes for cholesterol (*k* = 4) and heart conditions/illnesses (*k* = 8) may have limited power to detect associations for such outcomes, however this was not the case for the null finding between racism and blood pressure/hypertension (*k* = 24). In a recent meta-analysis Dolezsar et al. [34] also found that racism was not significantly related to either blood pressure or hypertension. Whereas previous meta-analyses in this field have tended to consider physical health outcomes as a group, our additional examination of disaggregated outcomes reveals mixed findings across distinct physical health outcomes. Although some research has suggested the relationship between racism and blood pressure may be curvilinear [121, 281, 304, 402–403], this possibility was not explored in the current meta-analysis due to limitations in the statistical analysis program we utilized.

The stronger association between racism and mental health outcomes, compared with physical health, raises questions about the mechanisms by which racism affects health. Chronic exposure to racism may be implicated in hypothalamic-pituitary-adrenal (HPA) axis dysregulation that, in turn, can damage bodily systems and lead to physical outcomes such as CVD and obesity. The impacts of racism on the dysregulation of cognitive-affective regions such as the prefrontal cortex, anterior cingulate cortex, amygdala and thalamus share similarities with pathways leading to anxiety, depression and psychosis [404]. Neuroimaging studies have also identified activation of these regions in response to social rejection that are correlated with self-report distress levels and are analogous to the activation of regions involved in physical pain [405]. Such neurobiological changes may also be precursors to racism-related vigilance and rumination which are emerging as health risk factors in their own right [406–411].

For negative mental health, the effect size for studies using cross-sectional data was larger than the effect size for studies using longitudinal data. We found that long-term longitudinal data (more than one year between exposure and outcome) showed weaker, although still significant, associations between racism and health compared to either cross-sectional or shorter-term longitudinal data (up to one year between exposure and outcome). Schmitt et al. [23] have found similar results with regards to discrimination more broadly. This finding suggests that the detrimental impact of racism may attenuate over time, perhaps because of the fading impact of brief exposure or due to individuals becoming ‘hardened’ to racism over time [412, 413]. The implications of such preliminary evidence for the etiological ‘half-life’ of racism warrant further investigation through a greater focus on longitudinal data in the field (comprising only 9% of articles to date).

Moderation by age, sex, education level and birthplace of study participants has been found in previous individual studies (e.g., [42, 414]) but has been inconclusive in previous systematic reviews and meta-analyses [e.g., [13]). Similarly, we found that these variables did not significantly moderate the association between racism and the health outcomes. Ethnicity, however, significantly moderated the association between racism and depression, negative mental health, and physical health, providing tentative evidence that the association between racism and negative mental health is stronger for Asian Americans and Latino/a Americans compared with African Americans, and that the association between racism and physical health was stronger for Latino/a Americans compared with African Americans. There were no significant differences between African Americans and either European or Native Americans. These findings could suggest that African (and possibly Native) Americans are more resilient to racism than other minority groups [415]. It is also possible that, as may be the case for European Americans [416], their experiences are qualitatively distinct from other minority groups. These results should be treated as preliminary given the number of studies reporting effects for some ethnic groups was rather small (for example, only 6 studies of Native Americans and 6 studies of European Americans were used in analyses of racism and mental health, and only 5 studies of Latino/a Americans were used in analyses of racism and physical health).

Other studies have found variations in correlation strength within Latino/a American groups (Cubans, Mexicans, Dominicans and Puerto Ricans) [29] and between Asian, African and Latino Americans in relation to chronic conditions [417]. The magnitude of associations in our meta-analysis was similar (or slightly higher) than findings from previous meta-analyses that focused on these specific ethnic/racial population groups [28–31].

In terms of study quality, there was some indication of publication bias whereby some associations between racism and health outcomes were stronger in published versus unpublished studies, consistent with the tendency for ‘null’ studies to remain unpublished (i.e., the ‘file drawer’ problem [418]). Similarly, studies using non-representative samples had larger effect sizes than those using representative samples, indicating that bias may be introduced through ‘convenience’ and other sampling strategies. This finding raises questions about the potential role of sampling bias. Convenience samples often employ strategies such as snowball sampling, whereby participants are connected to one another, which then leads to problems with autocorrelation, or recruitment via advertisements in community locations that could lead to recruitment of those participants for whom racism is more salient, thus inflating effect sizes. By contrast, larger and more representative studies often use more sophisticated sampling methods, as well as statistical corrections (e.g. sampling weights) that may lead to less biased estimates. We stress that the associations between racism and health are evident regardless of the study methodology, but note that studies using convenience may overestimate the association between racism and health outcomes.

It is not immediately clear why associations between racism and some negative mental health outcomes were stronger in studies published more recently (from 2006) or in non-U.S. studies. Further research, including meta-regression to control for potential methodological covariates, should investigate such trends more closely. Unlike Pieterse et al. [31] who found no moderation by sample, publication or instrument type in their meta-analysis of perceived racism and mental health for Black Americans, we found stronger associations for some health outcomes in studies utilizing direct rather than indirect exposure measures, exposure assessment with specified rather than unspecified timeframes, and instruments with more rather than less items. The latter resonates with previous findings that the association between racism and health is stronger in studies that employ multiple item or multiple domain measures of racism [10, 22, 419].

One of the key challenges in the study of racism and health is the profusion of exposure measurements currently utilized by researchers [420, 421]. Combining the eight most popular scales accounts for only about a third of extant articles, with the Schedule of Racist Events (the most commonly utilized tool) being referenced in only around 10% of articles. As the field matures, it is likely that measurement will converge on validated best-practice instruments. Both the Everyday Discrimination Scale [422] and Experiences of Discrimination scale [221] are likely candidates given applicability to a range of ethnoracial groups and extensive psychometric validation [280, 423–428], although a focus on how these and other instruments can validly assess racism among children and youth is currently lacking [14]. We caution however, that our focus on explicit mentions of race will tend to understate the contribution of scales like the Everyday Discrimination Scale, for which attributions to race are volunteered by participants, rather than intrinsic to the question asked.

This study noted some differential findings by exposure measures, for example for PRS. Studies using PRS found stronger effects of racism on mental health when compared with studies using RaLES, whereas the effect of racism on physical health was weaker in studies using PRS compared with studies using EDS and EOD. These novel findings should be investigated in future studies that explicitly compare effects between various measures (e.g., [429]).

Internal reliability, however, did not moderate associations between racism and health. Alpha coefficients are the most commonly reported indicator of a study’s reliability, but unfortunately, only measure a single dimension (internal consistency). More valid and reliable instruments should be better able to detect associations between racism and health. Accordingly, we encourage authors to provide more comprehensive description of their instrument’s psychometric properties (e.g., test-retest). Finally, the relatively weaker association found in studies using indirect measures (e.g., group and vicarious exposures to racism) may stem from the relatively under-developed nature of measurement approaches to date (e.g., [295, 430, 431]), highlighting a need for further development.

Although required to estimate variations in exposure over time [432], explicit time frames were not always included in exposure instruments. Gee, Walsemann and Brondolo [413] have argued that explicit attention to the timing of racism is critical both for theoretical and empirical reasons, especially these dimensions of time: (1) the length of exposure to discriminatory events; (2) the timing of these events within the life course; and (3) the etiological period between exposure and the onset of illness. While in this study the effects of racism on health were not modified by age (categorized as 18 years and older vs. less than 18 years), it is highly plausible that children are more vulnerable to the harmful effects of racism, and that experiences of racism in the early years of life have more severe and persistent health consequences than racism experienced later in life [14]. This is likely through the biological embedding of early life stress as well as weathering effects resulting from chronic exposure to stress throughout life [433]. The few studies on racism and health conducted among pre-adolescent populations limit the extent to which moderation effects by age can be tested. More longitudinal studies commencing early in the lifespan, from pre-conception onwards, are required to further elucidate these causal pathways.

This study includes a number of limitations. First, it does not include articles in languages other than English and thus may under-represent studies from countries which publish in other languages. Second, it focuses only on unadjusted associations, mainly because of the challenges related to adjusting for different sets of covariates. A recent meta-analysis demonstrated that for mental health and discrimination more generally, differences between unadjusted associations and those adjusted for covariates were not significant [23]. Nonetheless, we concur with Pascoe and Richman [13] that a specific set of control covariates should be reported in future studies to allow more thorough meta-analytic investigation of partial correlations. A growing body of literature may also allow further elucidation of moderators using meta-regression. Although beyond the scope of this paper, a fine-grained examination of individual outcomes (e.g., psychological stress, depression) is also required given evidence of differential associations between racism and various health outcomes.

This study is the most comprehensive meta-analysis on racism and health to date, providing information on the state of play in this rapidly growing field. Our findings corroborate previous research findings as to the magnitude of associations between racism and mental health, adding novel meta-analyses of associations between racism and a diverse range of outcomes, including overweight, somatization, psychological stress, and post-traumatic stress (PTS) and stress disorder (PTSD). It also provides evidence that racism has long-term effects on health that remain significant despite attenuation over time. It is hoped that this meta-analysis can provide new directions for research in understanding, as well as addressing, racism as a determinant of ill-health.

## Supporting Information

S1 Table PRISMA 2009 Checklist. (PDF)

S1 Appendix Search Strategy. (PDF)
