# Supplementary material for: Racism as a Determinant of Health: A Systematic Review and Meta-Analysis
Source: PLoS One. 2015 Sep 23;10(9):e0138511. doi: 10.1371/journal.pone.0138511 (PMC4580597; doi:10.1371/journal.pone.0138511)
Supplement: S1 Appendix — (PDF) [file pone.0138511.s002.pdf]

S1 Appendix. Search Strategy

MEDLINE Search Strategy (adapted for other databases as needed)

Terms:
1 birth* (ts)
2 gestation* (ts)
3 health* (ts)
4 well-being (ts)
5 wellbeing (ts)
6 disease* (ts)
7 illness* (ts)
8 BMI (ts)
9 “body mass index” (ts)
10 WHR (ts)
11 “waist hip ratio (ts)
12 anthropometric* (ts)
13 “blood pressure” (ts)
14 hypertension (ts)
15 cardiovascular (ts)
16 overweight (ts)
17 obes* (ts)
18 depressi* (ts)
19 anxi* (ts)
20 distress (ts)
21 stress (ts)
22 suicid* (ts)
23 sleep* (ts)
24 (social* OR behav* OR emotio* OR develop* OR psych*) (AB) AND (difficul* OR problem* OR delay* OR adjust*) (ts)
25 self-esteem (ts)
26 “self esteem” (ts)
27 “life satisfaction” (ts B)
28 “quality of life” (ts B)
29 resilien* (ts)
30 alcohol (ts)
31 tobacco (ts)
32 smok* (ts)
33 “substance use” (ts)
34 drug* (ts)
35 health (ts) AND (care OR service* OR clinic*) (ts)
36 Diseases (MESH)
37 Psychiatry (MESH)
38 Psychology (MESH)
39 1 - 38 (OR)
40 ts=(discrim* OR bias OR prejud* OR hostil* OR harass* OR bully* OR “unfair treat*” OR oppress*) OR mh=prejudice
41 ts=(rac* OR ethnic* OR cultur* OR religio* OR migra* OR immigra* OR refugee*) OR mh=ethn groups OR mh= minority groups

42 ts=(longit* OR cohort* OR trial* OR “follow up” OR prospective OR retrospective OR “cross section*” OR cross-section* OR intervention* or quantitative or survey* OR “case- control” or “case control” or “randomised control* trial*” or “randomized control* trial*” or ”before and after” or “interrupted time series” or questionnaire* or registr* OR evaluat* or audit*) OR mh=Longitudinal Studies OR mh=Epidemiologic Research Design OR mh=Epidemiologic Study Characteristics as Top[illegible] or mh=registries

43 39 AND 40 AND 41 AND 42
